# Automatic Premature Ventricular Contraction Detection Using Deep Metric Learning and KNN

**DOI:** 10.3390/bios11030069

**Published:** 2021-03-04

**Authors:** Junsheng Yu, Xiangqing Wang, Xiaodong Chen, Jinglin Guo

**Affiliations:** 1 School of Electronic Engineering, Beijing University of Posts and Telecommunications, Beijing 100876, China; jsyu@bupt.edu.cn (J.Y.); guojinglin@sgitg.sgcc.com.cn (J.G.); 2 Queen Mary, University of London, London E1 4NS, UK; xiaodong.chen@qmul.ac.uk

**Keywords:** electrocardiogram, deep metric learning, k-nearest neighbors classifier, premature ventricular contraction

## Abstract

Premature ventricular contractions (PVCs), common in the general and patient population, are irregular heartbeats that indicate potential heart diseases. Clinically, long-term electrocardiograms (ECG) collected from the wearable device is a non-invasive and inexpensive tool widely used to diagnose PVCs by physicians. However, analyzing these long-term ECG is time-consuming and labor-intensive for cardiologists. Therefore, this paper proposed a simplistic but powerful approach to detect PVC from long-term ECG. The suggested method utilized deep metric learning to extract features, with compact intra-product variance and separated inter-product differences, from the heartbeat. Subsequently, the k-nearest neighbors (KNN) classifier calculated the distance between samples based on these features to detect PVC. Unlike previous systems used to detect PVC, the proposed process can intelligently and automatically extract features by supervised deep metric learning, which can avoid the bias caused by manual feature engineering. As a generally available set of standard test material, the MIT-BIH (Massachusetts Institute of Technology-Beth Israel Hospital) Arrhythmia Database is used to evaluate the proposed method, and the experiment takes 99.7% accuracy, 97.45% sensitivity, and 99.87% specificity. The simulation events show that it is reliable to use deep metric learning and KNN for PVC recognition. More importantly, the overall way does not rely on complicated and cumbersome preprocessing.

## 1. Introduction

The heart is a vital part of the muscular system, which keeps blood circulating. Heart rhythm and heart rate are two fundamental indicators to assess whether the heart is working orderly [1]. Heart rhythm is usually rhythmic, and its clinical significance is more important than the heart rate. However, suppose the heart’s four chambers, including the right atrium (RA), right ventricle (RV), left atrium (LA), and left ventricle (LV), cannot alternately contract and relax to pump blood through the heart. In that case, the heartbeat will be abnormal in speed and rhythm. The irregular heartbeat typifies arrhythmia and harms the body’s organs and tissues, such as the lungs and brain [2]. Table 1 lists the most common types of arrhythmia.

**Figure 1:**
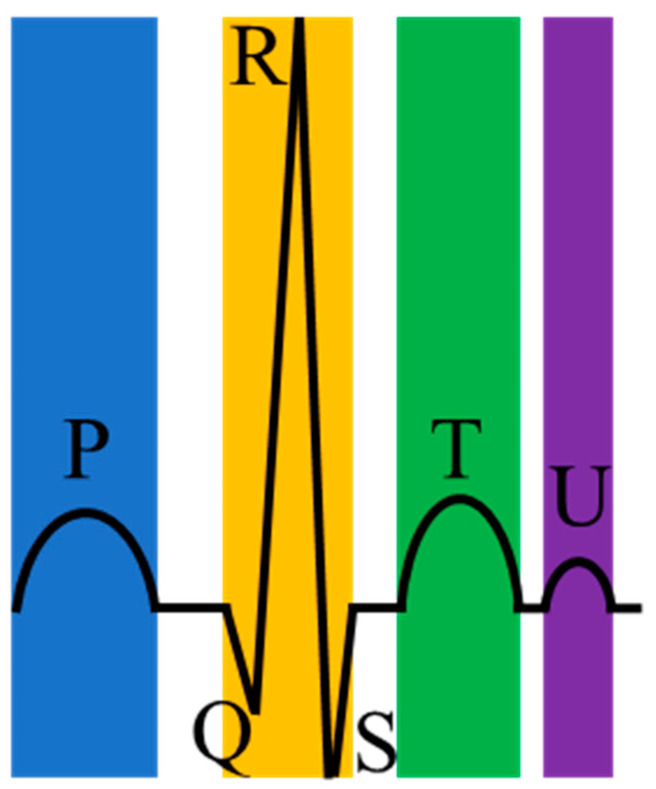
A normal heartbeat in an electrocardiogram (ECG).

Arrhythmias are closely related to electrical irregulars of the pumping heart [3]. Precisely, the heart’s electrical system controls the heartbeat by the electrical signal. However, when these electrical signals that should have traveled on a fixed path change or the heart tissue changes, arrhythmias occur. For most arrhythmias, the electrocardiogram (ECG) is a handy and visual tool and has the advantages of being simple, fast, and accurate [4]. ECG can record the heart’s electrical signals and is non-invasive and affordable for ordinary people. Moreover, a normal heartbeat in ECG has four main entities: A P wave, a QRS complex (a combination of the Q wave, R wave and S wave), a T wave, and a U wave, as shown in Figure 1. Table 2 shows the cause of generating these waves.

However, ECG is powerless for some particular arrhythmias, such as premature ventricular contraction (PVC), because the patient has a limited time for testing on the ECG machine during a standard ECG recording. PVC is a common arrhythmia initiated in the ventricles and often occurs in repeating patterns, as stated in Table 3. Specifically, PVC is ubiquitous in healthy individuals and patients and is associated with many diseases. There is a study evaluating the prevalence of frequent PVCs in Guangzhou, China [5]. Above 1.5% of the residents who received 12-lead ECG had PVCs, and nearly 1/6 of subjects who received 24-h Holter ECG were diagnosed with PVCs. According to the report provided by the American College of Cardiology Electrophysiology Council, PVC is related to left ventricular dysfunction and cardiomyopathy [6].

Furthermore, PVC is also associated with some disorders, such as ventricular tachycardia (VT), ventricular fibrillation (VF), underlying coronary artery disease, hypertension, and myocardial infarction (MI) [7,8,9]. Because PVC usually causes few or no symptoms, self-diagnosis is not accessible. Most people go to the hospital for help only after they notice severe symptoms.

Since the Holter monitor is a small wearable device and can record the heart’s behavior in the patient’s everyday life, cardiologists usually use the Holter monitor as a medium to obtain long-term ECG and diagnose PVC in clinical practice. However, analyzing so many long-term ECGs takes a lot of time and energy for cardiologists. Therefore, it is crucial to improve the efficiency of cardiologists regarding reliable and automatic searching for PVC from the long-term ECG.

With the continuous advancement of technology for collecting and processing physiological signals in recent years, many researchers have developed various algorithms to detect PVC from the long-term ECG automatically, as summarized in Table 4. In general, these algorithms are mainly of two types: Morphology-based methods and deep learning-based methods. In these morphology-based methods, extracting features relies on strong expertise, and most researchers have to manually design each feature to ensure that the features are practical. In the deep learning-based methods, extracting features is automatic, which is the most significant difference between the two methods.

**Figure 2:**
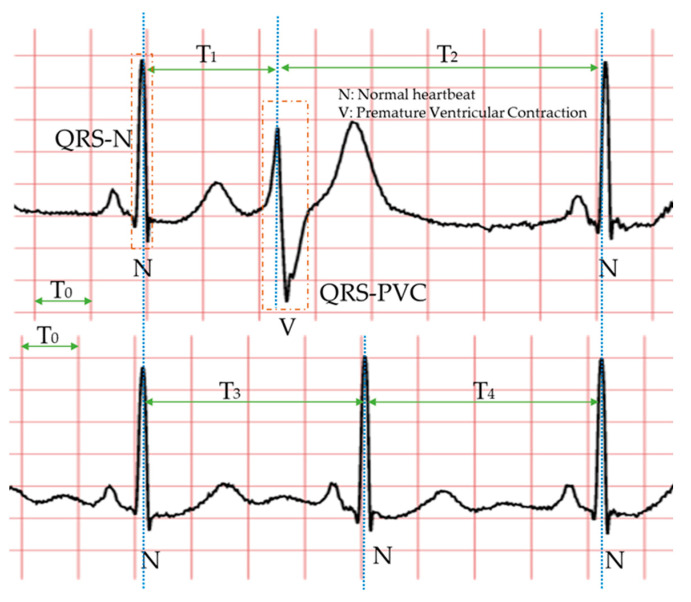
The waveforms of PVC and normal heartbeat. The two ECGs in this picture are from the same person. Each symbol is defined as follows. N (normal heartbeat); V (premature ventricular contraction); T_0_ (0.20 s); T_1_ (R-R interval); T_2_ (R-R interval); T_3_ (R-R interval); T_4_ (R-R interval); QRS-N (QRS complex of normal heartbeat); QRS-V (QRS complex of PVC). The important thing is that T_3_ and T_4_ are usually equal, and the sum of them is generally similar to the sum of T_1_ and T_2_. The blue dotted line indicates the location of the R wave peak in each heartbeat.

Specifically, the morphology-based method’s core is designing a series of trustworthy features manually with professional knowledge and experience. Compared with the normal heartbeat, PVC’s waveform usually has three main characteristics, as shown in Figure 2: The QRS complex is broad and has an abnormal morphology (QRS-N and QRS-V); it occurs earlier than expected for the next sinus impulse (T_1_ < T_3_ < T_2_); full compensatory pause (T_1_ + T_2_ = T_3_ + T_4_). Therefore, in the morphology-based methods, some classic features mostly come from the time-domain or frequency-domain of the ECG. Due to the continuous development of machine learning algorithms and the advancement of professional knowledge related to signal processing and ECG, most researchers have favored the morphology-based methods. Moreover, these approaches have occupied an unshakable status for a long time.

The signals, collected directly from wearable devices, are always noisy. These noises mainly include baseline wander, 60 Hz power-line noise, electromagnetic interference, 100 Hz interference from fluorescence lights, and motion artifacts. Therefore, many morphology-based methods usually denoise the long-term ECG to extract features more accurately. These popular denoising algorithms are usually based on filters [10,11,12] or wavelet transforms [13,14].

Secondly, the morphology-based methods design and extract a series of features according to the expertise related to ECG and signal processing. Adnane et al. proposed a vital feature based on the Haar wavelet transform coefficients [15]. Du et al. also recommended an essential feature obtained by the chaotic analysis and Lyapunov exponent, named the chaotic feature [16]. Lek-uthai et al. extracted the four features based on cardiac electrophysiology: R-R interval, pattern of QRS complex, width of QRS complex, and ST-segment (the end of the QRS complex to the beginning of the T wave) level [17]. Jenny et al. suggested using the independent component analysis (ICA) algorithm to extract features and applying *t*-test analysis to evaluate these features [18]. Nuryani et al. redefine the width and the gradient of the QRS wave and regarded them as features [19].

Another factor determining the PVC detection method’s performance is the classifier, which classifies samples with these extracted features. The essence of the classifier is a hypothesis or discrete-valued function. There are some popular classifiers used to distinguish regular and PVC beats: Artificial neural networks (ANN) [20,21,22], learning vector quantization neural network (LVQNN) [23], k-nearest neighbours (k-NN) algorithm [24,25], discrete hidden Markov model (DHMM) [26], support vector machine (SVM) [27,28], Bayesian classification algorithms [29], and random forest (RF) [30].

In summary, the morphology-based methods include three essential components: Denoising, designed features, and classifiers. Noise reduction is a prerequisite for accurately extracting features. Feature extraction is the core. The classifier directly plays a decisive role in the performance of these methods. Although the morphology-based methods have achieved significant success on this project after many researchers’ efforts, these methods still have some limitations. First, the process of feature extraction relies heavily on preprocessing, such as wavelet transform and QRS detection. Preprocessing undoubtedly increases computational overhead. Further, extracting features is a complex and professional process. In this process, features are not imagined out of thin air but based on knowledge and experience. The features in each literature are often different from person to person, which makes it biased. Therefore, some scholars have proposed deep learning-based methods, which can detect PVC without manually designing features.

Deep learning-based methods are also inseparable from denoising, designed features, and classifiers. Compared with the morphology-based methods, the deep learning-based methods usually do not require professional knowledge and experience related to ECG or signal processing to design features automatically. Although these features are challenging to understand intuitively, these features are useful. That is to say, in most cases, we do not know the meaning of these features, but these features can be used to distinguish between a normal heartbeat and PVC.

Conway et al. used an ANN to detect PVC without manually extracting features [31]. The ANN’s input corresponds to the 30 points of the QRS complex. Yang et al. proposed an innovative algorithm based on sparse auto-encoder (SAE) to extract features [32]. SAE is an unsupervised learning algorithm, including two processes of encoding and decoding. The encoding process performs the features’ extraction, and the decoding process ensures the effectiveness of the features. Zhou et al. suggested an approach based on the lead convolutional neural network (LCNN) and long short-term memory (LSTM) network to extract features [33]. Liu et al. proposed a PVC detection method, which can directly analyze and process the ECG waveform images [34]. The finetuned Inception V3 model, developed by Google, is the core component of the method [35]. 

It is worth noting that feature extraction and classification are closely connected and inseparable. Liu et al. also recommend using a one-dimensional convolutional neural network (1D CNN) to classify the ECG time-series data obtained from ECG waveform images. Zhou et al. reported a PVC detection method based on the recurrent neural network (RNN) [36], which has natural and inherent advantages in processing time-series signals because of its internal memory. Hoang et al. proposed a PVC detection model deployed in wearable devices [37]. The model is based on a CNN and can be scalable from 3-lead to 16-lead ECG systems.

The deep learning-based methods alleviate the limitations of morphology-based methods and have the following three advantages. (1) The deep learning-based methods can use specific network structures to extract features, such as the convolutional kernel. This process does not require human intervention. (2) In extracting features, the deep learning-based methods can continuously optimize features to ensure that the features are practical and non-redundant, such as pooling operation. (3) The deep learning-based methods are less affected by preprocessing, such as detecting and locating the QRS waveform.

However, these existing deep learning-based methods are not without flaws. Most of the features extracted by deep learning algorithms are difficult to understand intuitively. The performance of the deep learning-based methods is slightly inferior to the morphology-based methods, as shown in Table 4. Some deep learning-based methods need to preprocess the ECG. In the literature [36], much preprocessing is required before the model training, such as resampling, signature detection, and normalization. In addition, the research [37] takes 2D time-frequency images obtained by wavelet transform on the ECG as the proposed network’s input. No doubt preprocessing increases the computational overhead.

In summary, we can quickly draw the following conclusions according to the above discussion and Table 4. (1) Most of the methods mentioned in the literature are based on morphology. Table 4 lists 27 references, of which 22 belong to the morphology-based method, and only five belong to the deep learning-based method. (2) Most researchers prefer to use ANN, KNN, and SVM to identify PVC after completing the feature extraction. Six pieces of literature in Table 4 use ANN as a classifier. (3) The R-R interval is an excellent feature, which has been recognized by the majority of researchers. Nearly one-third of morphology-based methods have used this feature. (4) In terms of accuracy, sensitivity, and specificity, these three classifiers, FNN, BCM, and SSVM, achieved the best results, respectively. Overall, the morphology-based method’s performances were slightly better than deep learning, due to the expert’s knowledge and experience.

Consider the following: On the one hand, it is easy to understand the features extracted by the morphology-based methods, but feature engineering is the most significant limitation of this method; on the other hand, it is very difficult or even impossible to understand intuitively the features extracted by the deep learning-based methods, but deep learning algorithms can automatically extract and optimize features. This research proposed a novel approach based on deep metric learning and KNN to ensure that the features used to detect PVC can be extracted automatically and understood intuitively.

Specifically, the proposed method introduced deep metric learning into PVC inspection projects for the first time. It is worth mentioning that deep metric learning can automatically extract features, and these features are usually in the high-dimensional embedding space. In this case, the KNN classifier is undoubtedly an optimal choice. Second, the proposed method did not rely on expert knowledge and experience related to ECG, significantly reducing the threshold for studying physiological signals. In theory, the proposed method is suitable for the most physiological signals. Third, to improve the efficiency of detecting PVC from long-term ECG, this method can directly classify heartbeats. Preprocessing, such as denoising, is unnecessary. Finally, clinical ECG from the MIT-BIH (Massachusetts Institute of Technology-Beth Israel Hospital) Arrhythmia Database [38,39] evaluated and verified the proposed method’s performance and effectiveness. The following is the remainder’s arrangement: Section 2 describes the dataset, proposed framework, and evaluation measures; Section 3 presents and discusses the results; Section 4 gives the conclusion and directions.

## 2. Materials and Methods

### 2.1. Materials

In this paper, all ECG came from the MIT-BIH Arrhythmia Database, which plays an essential role as a referee in verifying arrhythmia detectors. The MIT-BIH Arrhythmia Database was first publicly released in 1980 and has been updated three times in 1988, 1992, and 1997. Its public release is a landmark event. Nearly one hundred research groups worldwide have used the MIT-BIH Arrhythmia Database in the eight years from the first release. Today, many academic and industrial researchers have affirmed the effectiveness of this database. Specifically, the MIT-BIH Arrhythmia Database contains 48 long-term Holter recordings obtained from 47 subjects: 25 men and 22 women. Every record is numbered from 100 to 234, with some numbers missing. Only records 201 and 202 are from the same male subject, and the remaining records corresponded to the other subjects one by one. Furthermore, each record contains two signals with a sampling rate of 360 Hz and a sampling duration of slightly over half an hour. 

In most records, the first signal is a modified limb lead II (MLII), and the second signal is usually a modified lead V1 (occasionally V2, V5, and V4). It is worth noting that at least two cardiologists independently annotate all signals in this database. Undoubtedly, free access to a large number of ECGs and beat-by-beat annotations through the internet at any time and anywhere has improved the efficiency of the development of arrhythmia detectors, which has been beneficial to numerous researchers. The ECGs used in this study were from the MLII, which appeared in almost all records. Considering the suggestion proposed by the Association for the Advancement of Medical Instrumentation (AAMI), this study discarded records 102, 104, 107, and 217 because of the paced beats. Furthermore, this research divided ECGs in the MIT-BIH Arrhythmia Database into the training set and test set, as shown in Table 5.

Notably, many datasets have adopted cross-validation to divide the training set and test set. However, applying cross-validation is unreasonable and may cause label leakage in this experiment. The reason is that the heartbeat of the subjects in the resting state hardly changes during a period of time. A reasonable division method should ensure that the same person’s ECG can not appear in both the training and test sets. Therefore, like most other studies, this study adopted the division method shown in Table 5, ensuring a reasonable comparison.

### 2.2. Methodology

**Figure 3:**
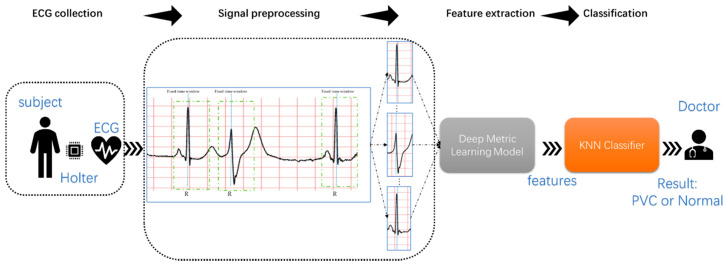
Block diagram of the proposed study.

Figure 3 shows the proposed method’s flow, namely, ECG collection, signal preprocessing, feature extraction, and classification. First, collecting long-term ECG is inseparable from wearable devices, such as Holter. Secondly, the proposed method extracted the single heartbeat from the MLII using a fixed time window and the R-peak detection algorithm. Then, the deep metric learning model could extract features of the heartbeat automatically. Finally, the KNN classifier predicted the category of the heartbeats based on the distance between the heartbeats. Since this research focused more on signal processing and analysis, the long-term ECGs and annotations came from the MIT-BIH Arrhythmia Database.

#### 2.2.1. Signal Preprocessing

Since the long-term ECG collected from the wearable device contained some noise, most existing research literature would use software algorithms to remove noise and baseline wander, such as the bandpass filter and wavelet transform. Considering that denoising increases the system’s computational load, the deep metric learning model can automatically extract features indicating the difference between the normal heartbeats and PVC heartbeats. Therefore, this study did not perform any operations related to denoising the signal but only segmenting the ECG.

The segmentation of ECG involves R-peak detection and a fixed time window. Specifically, the proposed method first applies the R-peak detection algorithm to locate the R-peak on the ECG. Because the existing R-peak detection algorithm [40,41,42,43] performs very well in accuracy and real-time, for example, Pan et al. designed an algorithm that can correctly detect 99.3% of the R-peak for the MIT-BIH Arrhythmia Database. This study directly used the MIT-BIH Arrhythmia Database’s R-peak position. 

Moreover, sliding a fixed time window on the ECG is a simple and straightforward way to obtain the same size’s heartbeats. In this research, the window’s length was 433. Each sliding should make the window’s vertical centerline coincide with each heartbeat’s R-peak. After these two steps, we could extract the normal heartbeats and PVCs from the ECG in each record.

#### 2.2.2. Feature Extraction

Feature extraction is an essential step for the development of PVC detectors. It is no exaggeration to say that the feature extraction defines the upper limit of the PVC detector. The classifier bounds how close the PVC detector is to its upper limit. For existing morphology-based methods, feature extraction is a complicated process. It relies heavily on feature designers’ knowledge and experience and reduces the efficiency of developing PVC detectors, because a set of excellent and efficient features often requires many researchers’ concerted efforts and a large number of experiments. 

Although deep learning-based methods can automatically extract features and avoid these limitations, the features, extracted through the classic network structures and optimization algorithms, are difficult to understand intuitively in these deep learning-based methods. Moreover, according to the existing literature, the deep learning-based methods’ overall performance is not significantly better than the morphology-based methods. It is particularly noteworthy that most of the methods suggested in the current literature have inadvertently ignored a severe issue that the number of normal heartbeats is much greater than PVC heartbeats in the MIT-BIH Arrhythmia Database.

Fortunately, the metric learning model can entirely solve the above problems. Metric learning is a type of mechanism to combine features to compare observations effectively. There are many types of metric learning models, such as stochastic neighbor embedding (SNE) [44], locally linear embeddings (LLE) [45], mahalanobis metric for clustering (MMC) [46], and neighborhood component analysis (NCA) [47]. The first two are unsupervised, and the latter two are supervised. Specifically, the metric learning model predicts the samples’ categories by measuring the similarity among samples [48]. Moreover, the model’s core is to establish a mapping function to represent the optimal distance metric.

Distinguishing features makes the classifier perform better. Metric learning is very good at extracting distinguishing features. Metric learning aims to make objects with the same label behave closer in the feature while increasing the distance between objects with different labels. To deal with various classification or clustering problems, we can select appropriate features through prior knowledge and experience on specific tasks. However, this method is very time-consuming, labor-intensive, and may also be unrobust to data changes. As an ideal alternative, metric learning can independently learn the metric distance function for a specific task according to different studies.

**Figure 4:**
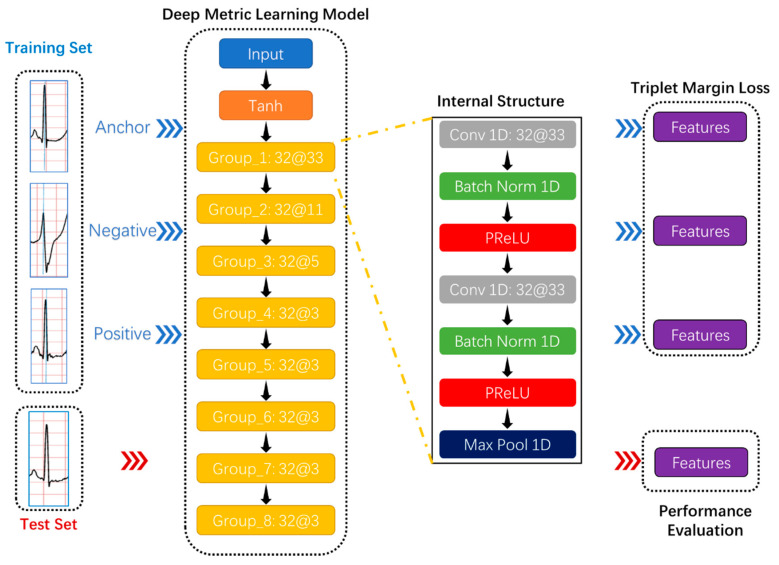
The proposed deep metric learning model’s basic architecture. Take “Group_1 32@33” as an example to comprehend the convolution group. “Group _1” is the convolution group’s name; “32@33” represents the number and size of the one-dimensional convolutional layer’s convolution kernels in the convolution group. Each convolutional group contains two 1D convolutional layers, two batch normalization layers, two activation functions, and one max-pooling layer.

Due to deep learning technology and activation functions, deep metric learning, as a combination of deep learning and metric learning, has provided excellent solutions in many classification tasks and attracted researchers’ attention in academia and industry. In the Humpback Whale Identification competition held on the Kaggle platform, which is the world’s largest data science community [49], the top five participating teams’ solutions all applied deep metric learning models: Triplet neural network [50] and siamese neural network [51]. The most conspicuous characteristic of these networks is the sharing weights, which makes the samples related because the triplet neural network can simultaneously learn both positive and negative distances and the number of training data combinations increases significantly to avoid overfitting. This study intended to use the triplet neural network as the deep metric learning model’s basic architecture, as shown in Figure 4.

Considering that the R wave peak is much larger than other points in the whole heartbeat, normalizing the heartbeat was beneficial to the deep metric learning model’s training. The Tanh function can normalize the input data between –1 and 1. Further, the Tanh function has little effect on real numbers close to 0 and has a more significant impact on real numbers far away from 0, especially these real numbers greater than one or less than −1. Equations (1) and (2) are the definitions of the Tanh function and its derivatives, respectively.

(1) $$\mathrm{Tanh}\left(x\right)\;=\;\frac{Sinh\left(x\right)}{Cosh\left(x\right)}\;=\;\frac{e^{x}-\;e^{-x}}{e^{x}+\;e^{-x}}$$

(2) $$\frac{dTanh\left(x\right)}{dx}\;=\;sech^{2}x=1-\;Tanh^{2}x$$

Secondly, the proposed deep metric learning model had eight convolutional groups that resulted in a feature vector representing a detected feature’s positions and intensity in the input data, as shown in Figure 4. Each convolutional group contained two 1D convolutional layers, two batch normalization layers, two activation functions, and one max-pooling layer. 

The 1D convolutional layer was the necessary component of automatic feature extraction. The purpose of the convolution operation was to extract different features of the input of this layer. In the entire network, the first few convolutional layers can usually only extract some low-level features. In contrast, the last layers can iteratively extract more complex features from the low-level features. The calculation of convolution was not complicated. The generated sequence could be obtained by repeating the following process: Move the convolution kernel in fixed steps along the input vector and calculate the dot product of the horizontally flipped convolution kernel and the input vector. The convolution definition is expressed as Equation (3), where x, h, y, respectively, represent the input vector, convolution kernel, and generated sequence.

(3) $$y_{j}=\;\overset{\infty }{\underset{i=-\infty }{\sum }}x_{i}\times h_{j-i}$$

Adding the batch normalization layer to the proposed deep metric learning model could improve the training efficiency by normalizing the convolutional layer’s feature map. When training the model, the batch normalization layer would sequentially perform the following operations [52]:

1. Calculate the mean and variance of the input vector;

(4) $$Batch\;mean\;\;\;\mu_{B}=\;\frac{1}{m}\overset{m}{\underset{i=1}{\sum }}x_{i}$$

(5) $$Batch\;\mathrm{variance}\;\;\;\sigma_{B}^{2}=\;\frac{1}{m}\overset{m}{\underset{i=1}{\sum }}{\left(x_{i}-\;\mu_{B}\right)}^{2}$$

2. Normalize the input using the mean and variance;

(6) $$\bar{x_{i}}=\;\frac{x_{i}-\;\mu_{B}\;}{\sqrt{\sigma_{B}^{2}+\;\epsilon }}$$

3. Attain the output with scaling and shifting;

(7) $$y_{i}=\;\gamma \bar{x_{i}}+\;\beta$$

In the Equations (4) and (7), m and ϵ, respectively, represent the number of samples per batch and a small constant for numerical stability. Further, γ and β are learnable parameters.

The rectified linear unit (ReLU) dramatically promoted the development of deep learning. Its use provided a better solution than that of the sigmoid function. The parametric rectified linear unit (PReLU) has improved ReLU and become the default activation function in many classification tasks [53]. Although PReLU introduces slope parameters, PReLU can better adapt to the other parameters like weights, and the increase in training costs is negligible. The mathematical definition of PReLU is Equation (8), where $y_{i}$ and $a_{i}$, respectively, represent the input on channel i and the negative slope which is a learnable parameter.

(8) $$f(y_{i})=\max\left(0,\;y_{i}\right)+\;a_{i}\times \min\left(0,y_{i}\right)$$

Adding the pooling layer to the proposed deep metric learning model could reduce the computational cost and effectively cope with the over-fitting by down-sampling and summarizing in the feature map. In addition, the pooling layer made the feature position change more robust, referred to by the “local translation invariance.” Three types of pooling operations have been widely used: Max-pooling, min-pooling, and average-pooling, as described in Table 6. However, the simultaneous use of min-pooling and PReLU would make each layer’s output results in the model almost all 0. Considering that the R wave waveform is sharp and high in a complete heartbeat, the max-pooling operation was applied in this study’s pooling layer.

Thirdly, training neural networks are inseparable from the loss function. The loss function can evaluate neural networks’ performance and play an essential part during training. The triplet margin loss [54] is used for measuring a relative similarity between samples. In this study, the triplet margin loss based on the cosine similarity calculated the model error required in an optimization process used to train the proposed deep metric learning model. Furthermore, the loss function for each sample in the mini-batch is:

(9) $$L\left(a,p,n\right)=\max\left\{d\left(a_{i},p_{i}\right)-d\left(a_{i},n_{i}\right)+margin,\;0\right\}$$

where

(10) $$d\left(\vec{x},\vec{y}\right)=\vec{x}\cdot \vec{y}=\left|\vec{x}\right|\left|\vec{y}\right|\cos\theta$$

The anchor, positive example, and negative example were three feature vectors and composed a triplet. Further, to make the model’s training process faster and more stable, applying the miner based on multi-similarity [55] could generate more valuable triplets. The multi-similarity contained three similarities in the general pair weighting (GPW) framework: Self-similarity, negative relative similarity, and positive relative similarity. In this study, the miner based on multi-similarity implemented the following process: Select a negative pair for the anchor if its similarity satisfies Equation (11); select a positive pair for the same anchor if its similarity satisfies Equation (12). Repeat the above steps with the feature vector obtained from each heartbeat as an anchor to obtain the index sets of its selected positive and negative pairs. These index sets are the basis of triples.

(11) $$S_{ij}^{-}>\underset{y_{k}=y_{j}}{\min}S_{ik}-\epsilon$$

(12) $$S_{ij}^{+}<\underset{y_{k}\neq y_{j}}{\max}S_{ik}+\epsilon$$

(13) $$S_{ij}=f\left(x_{i};\theta \right)\cdot f\left(x_{j};\theta \right)$$

In Equations (11)–(13), assume $x_{i}$ is an anchor, $y_{i}$ is the corresponding label, f is a neural network parameterized by θ, and · denotes the dot product, where $S_{ij}$ and ϵ, respectively, represent the similarity of two samples and a given margin.

#### 2.2.3. Classification

The classifier is the last link of the method proposed in this article and directly determines the classification system’s performance. In other research projects, the choice of classifier often depends on the results of multiple experiments. In other words, choosing a classifier requires many repeated experiments and costs much time. Many researchers often do experiments on several commonly used classifiers, such as SVM and ANN. Further, there is no reliable theoretical basis or clear direction to determine which type of classifier to use in most cases. Even if the researcher has determined which specific classifier to use, it is a huge challenge to adjust this classifier’s parameters.

However, in this article, since the features extracted by the deep metric model contain distance information, the KNN classification algorithm was the most suitable classifier. KNN classification algorithm is a type of non-generalizing learning. Unlike other classifiers that try to train a general model, the KNN classifier focuses on the distance. Moreover, the classification basis of the KNN is intuitive. The KNN classifier has only one parameter to control the number of votes, called *K*. The KNN classification algorithm first calculates the distance between the test data and each training data. If *K* is 1, the training data label with the closest distance is regarded as the predicted label. If *K* is greater than 1, the KNN classification algorithm votes according to the the top *K* training data labels with the smallest distance and finally determines the predicted label.

### 2.3. Evaluation Measures

The confusion matrix is a standard format for evaluating classification performance, and it usually appears in the form of a matrix. In most classification tasks, the confusion matrix summarizes the number of correctly and incorrectly predicted samples and those broken down by each class, providing researchers with a global perspective to comprehensively and efficiently evaluate the classifier’s performance, especially in imbalanced datasets. 

This study used the confusion matrix to measure the recognition performance of the proposed method. Further, this study used five evaluation indicators: Accuracy (ACC), sensitivity (Se), specificity (Sp), positive prediction (P_+_), and negative prediction (P_−_), based on the confusion matrix to compare more conveniently with experimental results in other literature. The confusion matrix and other five indicators, which also have been used in the literature [28], can be expressed as Equations (14)–(19). TN, FN, TP, and FP represent true negatives, false negatives, true positives, and false positives.

(14) $$\mathrm{Confusion}\;\mathrm{Matrix}\;=\;\left[\begin{array}{cc} \mathrm{TN} & \mathrm{FP}\\ \mathrm{FN} & \mathrm{TP} \end{array}\right]$$

(15) $$\mathrm{Accuracy}\;\mathrm{Acc}\;=\;\frac{\mathrm{TP}+\mathrm{TN}}{\mathrm{TP}+\mathrm{TN}+\mathrm{FN}+\mathrm{FP}}$$

(16) $$\mathrm{Sensitivity}\;\mathrm{Se}\;=\;\frac{\mathrm{TP}}{\mathrm{TP}+\mathrm{FN}}$$

(17) $$\mathrm{Specificity}\;\mathrm{Sp}\;=\;\frac{\mathrm{TN}}{\mathrm{TN}+\mathrm{FP}}$$

(18) $${\mathrm{Positive}\;\mathrm{prediction}\;P}_{+}\;=\;\frac{\mathrm{TP}}{\mathrm{TP}+\mathrm{FP}}$$

(19) $${\mathrm{Negative}\;\mathrm{prediction}\;P}_{-}\;=\;\frac{\mathrm{TN}}{\mathrm{TN}+\mathrm{FN}}$$

## 3. Results and Discussion

In this study, the main factors affecting the proposed system’s performance were as follows: The denoising method, the number of features, type of pooling layer, the loss function configuration, and type of classifier. First, denoising is a double-edged sword in the signal preprocessing stage. Denoising can improve the signal’s quality, reducing the difficulty of training a deep metric learning model. However, the signal may also lose some valuable information because of denoising.

Second, as a bridge between the deep metric learning model and classifier, the number of features is an essential hyper-parameter. This value cannot be too large or too small. The greater the number of features, the easier the features become redundant. Conversely, if there are too few features, the less information the features contain cause the classifier’s performance to deteriorate. Third, the type of pooling layer determines how features are summarized and retained and has the effect of de-redundancy. A proper pooling layer can select the most practical features to speed up the deep metric learning model’s training speed. 

Fourth, the loss function configuration is the top priority of training the deep metric learning model. The loss function and the miner based on multi-similarity cooperated in the proposed system. In the loss function, the margin should be within a reasonable range. The larger the margin, the more valuable the feature, but the harder it is to train the deep metric learning model. Conversely, the smaller the margin, the easier it is to train the model, but the less practical the features. Finally, the KNN classifier is hugely suitable for processing the deep metric learning model’s features. However, the choice of *K* value is highly dependent on the distribution of features. 

In this section, this study strictly divided the training set and the test set according to Table 5 and used them in each experiment. Before anything else, we evaluated the necessity of signal denoising. Secondly, we assessed the impact of the number of features on the proposed model. Immediately afterward, we tested pooling layers’ influence on the feature extraction of deep metric learning models. To improve the proposed system’s performance, we have adjusted the loss function and the miner parameters many times. Subsequently, we checked the performance of the KNN classifier and further optimized the classifier. Finally, we compared the proposed method with other research literature on multiple evaluation indicators, such as accuracy, sensitivity, and specificity. We carried out the simulation process on a Linux server with an Nvidia GeForce RTX 2070 GPU.

### 3.1. Experiment 1: Evaluation of the Necessity for Signal Denoising

In collecting ECG, wearable devices also collect noises. These noises can affect the quality of the signal and even distort the signal. The analog-to-digital conversion chip is a critical hardware component in wearable devices, directly determining the signal quality. Therefore, in the signal acquisition phase, researchers usually improve the sensor’s hardware equipment to suppress noise as much as possible. On the other hand, most scholars use software algorithms in the signal preprocessing stage to remove noise further. However, it is worth mentioning that the noise reduction algorithm inevitably changes the signal more or less. For the metric learning model used in this paper, the convolutional layer can automatically extract useful features and ignore useless information, such as noise. Therefore, the necessity of denoising the signal in the preprocessing stage is worth exploring.

Considering that the data used in this article were all from the MIT-BIH Arrhythmia Database and the method proposed in this paper focused on signal analysis, the denoising methods only involve software algorithms in the signal preprocessing. Expressly, we set up a set of comparative experiments to evaluate the necessity of signal denoising. This comparative experiment first processes and classifies the ECG directly according to the method proposed in this article, without applying any denoising means. Secondly, based on the first experiment, we only added some denoising algorithms in the signal preprocessing stage. 

**Figure 5:**
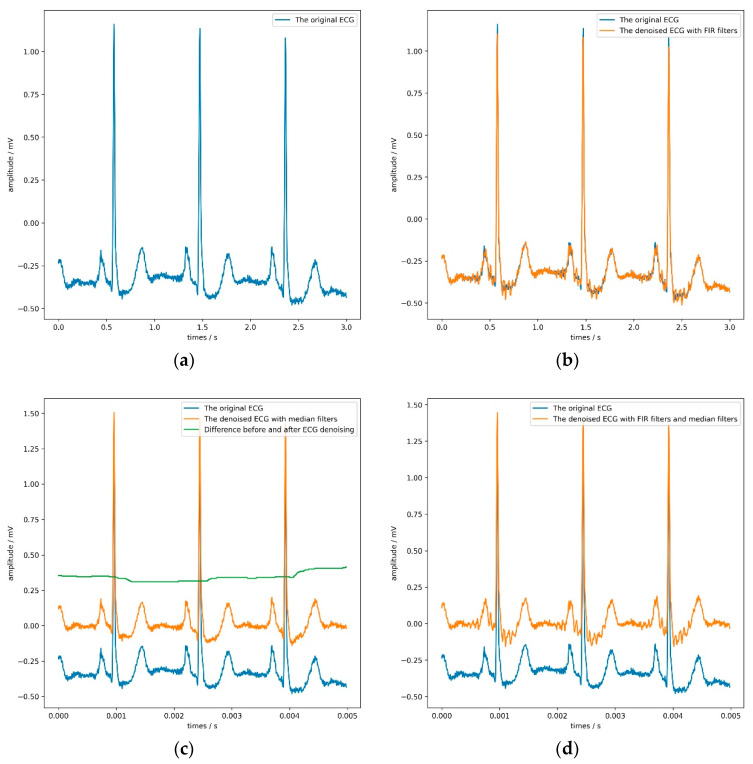
The result of applying different denoising algorithms on the ECG. (**a**) shows a 3-s ECG without denoising; (**b**) and (**c**) illustrate the effect of using finite impulse response (FIR) filters and median filters on the ECG, respectively; (**d**) shows the impact of using FIR filters and median filters on the ECG.

These denoising algorithms include two finite impulse response (FIR) filters with a sampling rate of 1000 Hz and two median filters. Figure 5 shows the denoising effect of the ECG. The former can filter 60 Hz power-line noise and 100 Hz interference from fluorescence lights, and the latter can remove the baseline of the signal and some noise. It is worth noting that the sizes of these two median filters window are 71 and 215, respectively, which is the same as the setting in literature [56]. Table 7 and Table 8 record the parameters and results of the comparative experiment in detail. In Table 7, the LR, WD, and *K* refer to the learning rate, weight decay, and the KNN classifier’s parameter.

It is not difficult to find from Table 8 that both the FIR filter and the median filter interfered with the model’s judgment to a certain extent, especially when applying both filters at the same time. Adding FIR filters and median filters in the signal preprocessing stage reduces each evaluation index of the model. The median filter can maximize the model’s sensitivity, but the model’s accuracy would drop slightly. According to the model’s overall performance, the most appropriate choice was not to use FIR filters or median filters. Figure 5 directly confirms this conclusion. 

By observing the four sub-pictures in Figure 5, we can quickly and intuitively discover two phenomena. First of all, the FIR filters could filter out specific frequency components but make the ECG show more obvious glitches simultaneously, which would be counterproductive. Second, the median filters could effectively remove the baseline but slightly change the ECG’s contour, which would be hidden danger for the model’s judgment.

According to Table 8 another thing worth noting is that the time required to process a half-hour-long ECG using the FIR filter and the median filter was 0.23 and 6.58 s, respectively, in this experiment. If this experiment used the computer hardware equipment with a lower frequency, the time spent on noise removal would become longer.

Considering the actual situation and experimental results, the method proposed in this paper had a particular anti-noise ability. Therefore, denoising was an option in this experiment’s signal preprocessing stage, though not a necessary option. Since this article focused on the classification of electrocardiograms, no more detailed research was done on noise reduction methods.

### 3.2. Experiment 2: The Choice of the Number of Features

There is no doubt that features are essential and directly determine the performance of the classifier. In theory, practical features should be informative, differentiated, and independent. The deep metric learning model can automatically extract features. In the process of producing high-quality features, the number of features is a critical parameter.

Suppose the number of features is too small. In that case, the deep metric learning model’s training process would be challenging. The acquired features are indistinguishable, and the information contained in the features is not enough to smoothly train the classifier.

On the contrary, too many features are redundant and increase the deep metric learning model’s training time. Further, the excessive features have the following shortcomings for the classifier: Expanding the classifier’s complexity, causing the dimensional disaster, and resulting in ill-posed problems and sparse features problems. These disadvantages eventually lead to a decline in the performance of the classifier.

Considering the above points, we conducted a series of experiments to find the appropriate number of features. We set different values for the number of kernels in the last convolutional layer to adjust the features. Table 9 provides the detailed results. Moreover, this experiment also adopted the basic configuration in Table 7.

**Figure 6:**
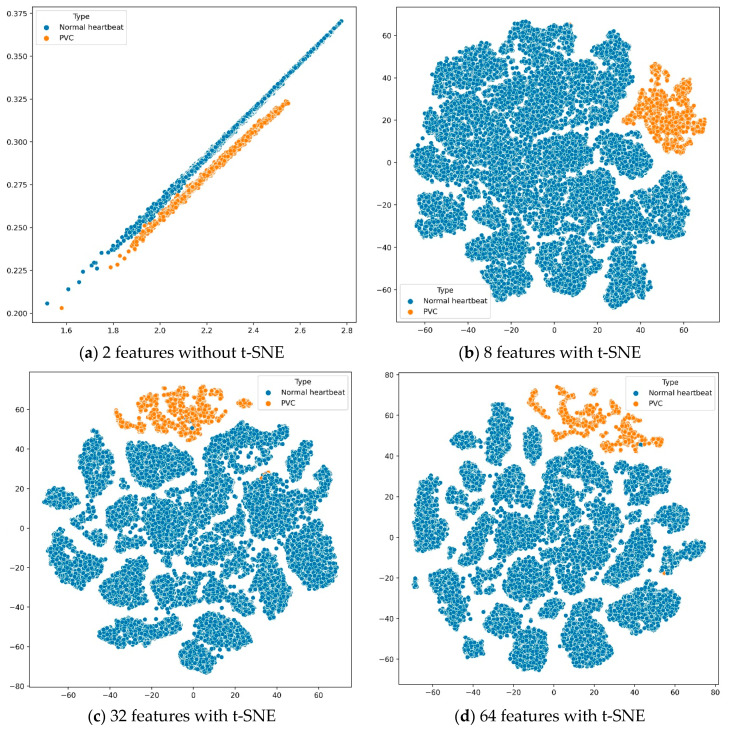
Visualizing the features of training data.

According to the experimental results in Table 9, we found that the features extracted by the deep metric learning model could make the KNN classifier perform best when the number of features was 32. Further, the number of features and sensitivity were positively correlated. In other words, the more features, the more confident the proposed system was in PVC predictions. To better analyze these results, we used *t*-distributed stochastic neighbor embedding (t-SNE) [57] to reduce the features’ dimension and then visualize the features in Figure 6. The t-SNE is a machine learning algorithm for dimension reduction, which is very suitable for reducing high-dimensional data to 2 or 3 dimensions for visualization.

Suppose we used the deep metric learning model to extract only two features. In that case, we could directly draw the features in a two-dimensional coordinate system without dimension reduction by t-SNE. When the number of features was greater than 2, we would use the t-SNE algorithm to reduce the features’ dimensions and display them on a two-dimensional plane. The four sub-images in Figure 6 show the distribution of different quantity features, extracted from all training data through the deep metric learning model, on a two-dimensional plane.

First, as shown in subfigure (a), the normal heartbeats and PVC were distributed on two parallel straight lines. However, when the first feature was around 2.1 and the second feature was around 0.26, the boundary between the normal heartbeat and PVC was not stark. Secondly, the other three subfigures showed that these features had obvious boundaries on the two-dimensional plane, distinguishing between the normal heartbeats and PVC. Finally, although the results in Table 9 are not much different, it is better to use the deep metric learning model to extract 32 features after comparing evaluation indicators such as accuracy and sensitivity.

### 3.3. Experiment 3: Assess the Impact of Pooling Type

In the CNN architecture, most researchers tend to insert a pooling layer in-between consecutive convolutional layers periodically. On the one hand, the pooling layer reduces the number of parameters to learn, avoiding over-fitting, and accelerating the deep metric learning model’s training speed. On the other hand, unlike the convolutional layer that extracts features with precise positioning, the pooling layer summarizes the features generated by a convolution layer, making the deep metric learning model more robust to variations in the position of the features in the input ECG. In other words, the pooling layer has a natural advantage in analyzing heartbeats of different cycles, even if these heartbeats come from different people.

Generally speaking, the core of the pooling layer is a fixed-shaped window. According to a set stride, this window slid overall feature regions and computed a single output for each location. It is worth noting that the way the pooling layer computes the output has no kernel and is deterministic, typically based on the maximum or average value of the features in the pooling window.

**Figure 7:**
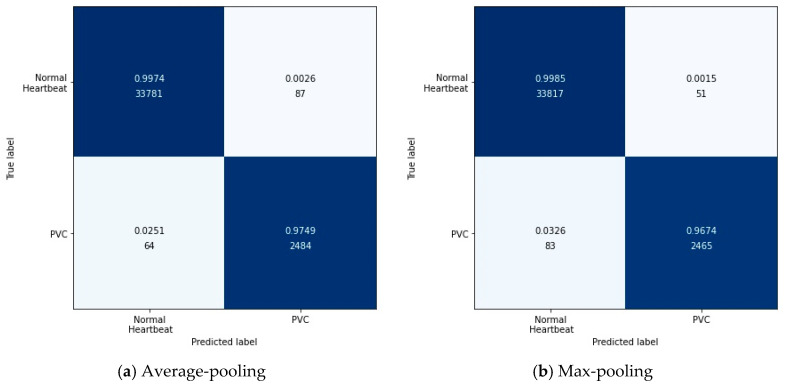
The confusion matrix about testing the pooling layer.

Specifically, the output after the max-pooling layer would contain the previous feature map’s most prominent features, which guarantees that each feature used to transmit to the next layer is practical. The average-pooling gives the average of features, taking into account global features in the pooling window. Therefore, in this experiment, we tested these two pooling layers’ performances in feature extraction with the configuration in Table 7. Figure 7 shows the results of this experiment in the form of a confusion matrix. Table 10 illustrates the detailed results in each evaluation index.

According to Figure 7, it can be found intuitively that the deep metric learning model with the max-pooling layer misjudged 134 test data, 17 fewer than the model with the average-pooling layer. Although the two models’ performances were similar, the max-pooling layer model was better at predicting normal heartbeats. The model with the average-pooling layer was more confident in predicting PVC, as shown in Table 10.

In the proposed method, feature extraction’s error mainly came from two aspects: The pooling window size and the feature shift caused by convolutional layer parameters. Generally speaking, the average-pooling operation could reduce the former error to preserve more information in the pooling window. The max-pooling operation can reduce the latter error to focus on the highest intensity information.

Since the loss function was based on cosine similarity, the desired model used to extract features should make the cosine similarity between samples of different classes as small as possible. Suppose the number of features was 2. The PVC and normal heartbeat features should be as close as possible to the two coordinate axes, respectively, in a two-dimensional coordinate system. Under careful consideration, the max-pooling layer was better than the average-pooling layer.

### 3.4. Experiment 4: Configure the Parameters of the Loss Function and Miner

In the triplet margin loss, the margin is an indispensable parameter that directly affects training the deep metric learning model. The definition of margin is the desired difference between the anchor-positive distance and the anchor-negative distance. Generally speaking, the larger the margin, the higher the quality of the extracted features. However, a large margin makes the model’s training process very unstable, and the loss makes it challenging to approach zero.

Secondly, in this paper, when we trained the model using the triples format’s training data, there were countless triples. However, since some triples met the margin requirements in the loss function, these triples did not contribute to the training model. There is no doubt that blindly and directly using all triples is time-consuming and inefficient for training models.

Fortunately, the miner based on multi-similarity can solve this problem. In this miner, epsilon is an important parameter that controls which triples are selected to train the model. Generally speaking, the larger the epsilon, the more triples are involved in training the model. To maximize the deep metric learning model’s performance, we conducted a series of experiments on margin and epsilon values with the configuration in Table 7. Table 11 lists the results for different values of margin and epsilon.

First of all, Table 11 shows that specificity and margin are negatively correlated, provide epsilon is 0. When the margins were 0.2, 0.4, 0.8, the proposed PVC detection system reached an accuracy of about 99.64% in these three experiments. However, when the margin was 0.1, the proposed PVC detection system performed best in the following indicators: Accuracy, specificity, and positive prediction. Secondly, increasing epsilon made the system’s overall performance worse, especially accuracy and positive prediction.

For the same batch of training data, the greater the margin, the greater the loss. In the case of a fixed learning rate, an enormous loss makes it difficult for the optimizer to find the best point, which leads to a decline in the quality of the extracted features. On the other hand, epsilon determines the number of triples involved in training. The larger the epsilon, the greater the number of triples in the same batch of training data, which undoubtedly increases the computational load. Furthermore, although the larger epsilon increases the number of triples, most of the triples can only produce a minimal loss, which leads to a reduction in the batch loss. A small loss may cause the optimizer to fall into a local optimum. Therefore, according to the experimental results, it is suitable to set the margin and epsilon to 0.1 and 0, respectively.

### 3.5. Experiment 5: Optimization of KNN Classifier and Comparison with Other Literature

In this article, the KNN classifier is suitable thanks to the spatiality of the features extracted by the deep metric learning model. Nevertheless, the performance of the KNN classifier is very dependent on the *K* value. A small *K* value is likely to cause overfitting, while an immense *K* value is likely to overlook some useful information in the training data. Therefore, it is necessary to test the *K* value. Table 12 lists the performance of the KNN classifier under different *K* values.

**Figure 8:**
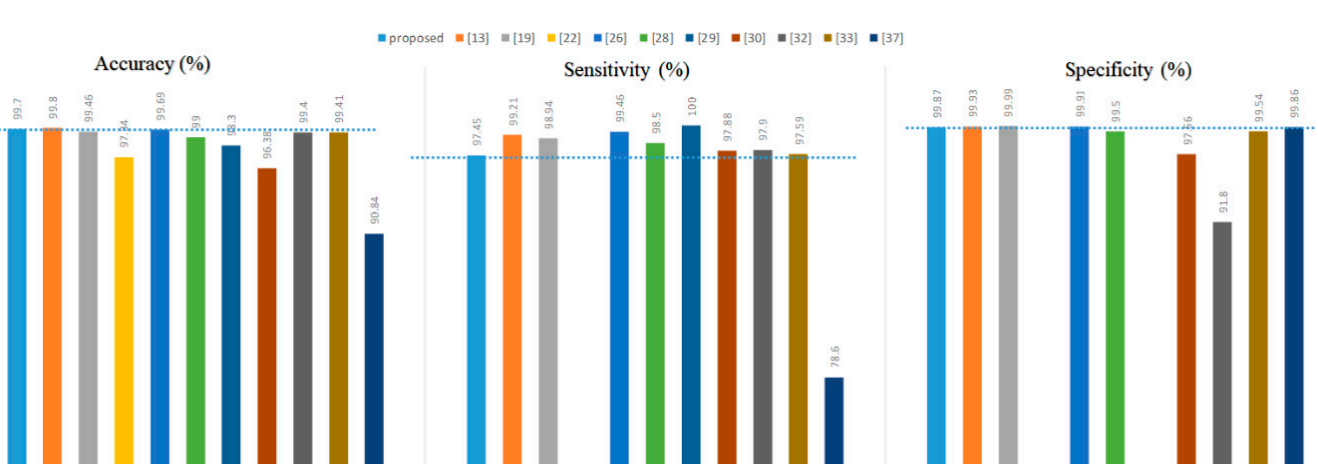
Comparison with other literature.

Overall, the best value of *K* was 1, which made the classifier obtain the highest accuracy. Secondly, as the *K* value continued to increase, the number of misjudgments by the KNN classifier for PVC was rising since the number of normal heartbeats was much larger than that of PVC. Finally, all the experimental results in Table 12 confirmed the effectiveness of the PVC detection method proposed in this article. Finally, we compared the proposed method with other literature, as shown in Figure 8.

As a whole, the proposed method was not superior in terms of accuracy, specificity, or sensitivity compared to the references [13,19,22,26]. However, they used long-term ECGs with no more than ten records from the MIT-BIH Arrhythmia Database to experiment. For example, references [19,22] used only six and five patient ECGs, respectively. In addition to this, randomly dividing the training set and test set should attract our attention and vigilance. For example, reference [26] randomly divided the training set and the test set at a ratio of 2:1. References [19,22] are no exception to this problem. It is particularly noteworthy that the training set and the test set were the same in reference [13], making their results unconvincing.

Second, the proposed method was only 0.1% lower than the reference [13] in accuracy and outperformed the others. In terms of specificity, the proposed method was also only inferior to reference [13,19,22,26]. However, this paper’s proposed PVC detection system did not perform exceptionally well in terms of sensitivity.

It is worth mentioning that the results of reference [28] were based on five-fold cross-validation. Suppose the division scheme of the training and test sets mentioned in this paper were used in reference [28]. In that case, the accuracy, sensitivity, and specificity of reference [28] would be 97.6%, 72.1%, and 99.9%, respectively. Therefore, our proposed method was superior to reference [28,29] used ten-fold cross-validation to select the classifier. Cross-validation is not suitable in the PVC detection task because it lays a hidden danger for label leakage. Further, our method did not rely on complex preprocessing and was superior to reference [37] in all metrics. Finally, the proposed system’s sensitivity was similar to that of reference [30,32,33]. Our method was superior to the methods presented in these three literature pieces in terms of accuracy and specificity.

In summary, our method outperformed other studies. Further, applying deep metric learning can automatically extract features and ensure that the features are spatially informative. Finally, the PVC detection system proposed in this paper was highly portable. The system could be directly applied to analyze many other physiological signals.

## 4. Conclusions

This study successfully applied a deep metric learning model to extract spatial features from heartbeats. These features were useful and practical. Moreover, the KNN classifier could directly classify heartbeats based on the distance between features. This paper’s series of experimental results showed that the proposed method achieved significantly better classification results than the existing morphology-based and deep learning-based methods. It was also practical and easy to migrate the proposed method to other physiological signals, such as heart sounds and pulses. Third, in this paper, we developed cosine similarity-based features. There were many other types of distance features to be developed. We plan to develop deep metric learning models based on different types of distances in future work to extract features. Combining multiple features helped to improve the performance of the proposed system. Finally, deploying the proposed method on cloud servers is in our plan, which will be of great help to patients and physicians in remote areas.

## Figures and Tables

**Table 1:** The most common types of arrhythmia.

Type	Characteristic
Tachycardia	Heart rate over 100 beats per minute
Bradycardia	Heart rate below 60 beats per minute
Supraventricular arrhythmias	Arrhythmias that begin in the heart’s upper chambers (atrium)
Ventricular arrhythmias	Arrhythmias that begin in the heart’s lower chambers (ventricles)
Bradyarrhythmias	Arrhythmias that caused by a dysfunction in the cardiac conduction system

**Table 2:** The cause of generating each wave in ECG.

Wave	Cause
P wave	Depolarization of the atrium
QRS complex	Depolarization of the ventricles
T wave	Repolarization of the ventricles
U wave	Repolarization of the Purkinje fibers

**Table 3:** The patterns of premature ventricular contraction (PVC) occurrence.

Patterns	Description
Bigeminy	Every other beat is a PVC
Trigeminy	Every third beat is a PVC
Quadrigeminy	Every fourth beat is a PVC
Couplet	Two consecutive PVCs
NSVT	Three-thirty consecutive PVCs

**Table 4:** Some algorithms for detecting PVC.

Reference	Features	Classifier	Accuracy	Sensitivity	Specificity
[10]	Eight features based on RQA	KNN and PNN	92.25%	73.33%	94.74%
[11]	Template-matching procedures	Threshold method	98.2%	93.1%	81.4%
[12]	12 features based on FT	ANN	98.54%	99.93%	98.3%
[13]	8 generalized wavelets transformed coefficients	FNN	99.8%	99.21%	99.93%
[14]	10 ECG morphological features and one interval feature	MLP	95.4%	-	-
[15]	Wavelet detail coefficients	Threshold method	98.48%	97.21%	98.67%
[16]	Chaotic feature	Threshold method	99.1%	93.6%	-
[17]	R-R interval, pattern of QRS complex, width of QRS complex, and ST-segment level	Main parameters algorithm	-	97.75%	98.8%
[18]	Using the ICA algorithm to extracts features	K-means and fuzzy C-means	80.94%	81.1%	80.1%
[19]	The width and gradient of the QRS wave	SSVM	99.46%	98.94%	99.99%
[20]	R-R interval and QRS width	ANN	96.29%	94.58%	96.59%
[21]	R-peak, R-R, QRS, VAT, Q-peak, and S-peak	ANN	99.41%	96.08%	-
[22]	R-R interval, QS interval, QR amplitude, and RS amplitude	ANN	97.34%	-	-
[23]	Feature extraction of Lyapunov exponent curve	LVQNN	98.9%	90.26%	92.31%
[24]	Using the PCA, SOM, ICA algorithm to extracts features	KNN	99.63%	99.29%	99.89%
[25]	Four morphological characteristics	DHMM	96.59%	97.57%	96.85%
[26]	Feature selection with GA	KNN	99.69%	99.46%	99.91%
[27]	Form factor and R-R interval	SVM	95%	-	-
[28]	A set of geometrical features	SVM	99%	98.5%	99.5%
[29]	80 features based on DFT	BCM	98.3%	100%	
[30]	R-R interval, R amplitude, and QRS area	RF	96.38%	97.88%	97.56%
[31]	Resampled QRS waveform	ANN	95%	-	-
[32]	20-dimensional feature vector obtained by using SAE	ANN	99.4%	97.9%	91.8%
[33]	Learned features automatically	LCNN, LSTM, and rules inference	99.41%	97.59%	99.54%
[34]	Learned features automatically	1D CNN and 2D CNN	88.5%	-	-
[36]	Learned features automatically	RNN	96–99%	99–100%	94–96%
[37]	Learned features automatically	2D CNN	90.84%	78.6%	99.86%

Abbreviations: Recurrence quantification analysis (RQA), Fourier transform (FT), independent component analysis (ICA), principal component analysis (PCA), self-organizing maps (SOM), genetic algorithm (GA), discrete Fourier transform (DFT), sparse autoencoder SAEK-nearest neighbor (KNN), probabilistic neural network (PNN), artificial neural networks (ANN), fuzzy neural network (FNN), multilayer perceptron (MLP), support vector machine (SVM), swarm-based support vector machine (SSVM), learning vector quantization neural network (LVQNN), discrete hidden Markov model (DHMM), Bayesian classification models (BCM), random forest (RF), lead convolutional neural network (LCNN), long short-term memory network (LSTM), one-dimensional convolutional neural network (1D CNN), two-dimensional convolutional neural network (2D CNN), recurrent neural network (RNN). Further, “-” means that relevant information is not mentioned in the literature.

**Table 5:** Dividing ECG into a training set and test set.

Dataset	Records	Normal Heartbeat	PVC
Training set	101, 106, 108, 109, 112, 114, 115, 116, 118, 119, 122, 124, 201, 203, 205, 207, 208, 209, 215, 220, 223, 230	35,640	2851
Test set	100, 103, 105, 111, 113, 117, 121, 123, 200, 202, 210, 212, 213, 214, 219, 221, 222, 228, 231, 232, 233, 234	33,868	2548

In this table, “Records” represents ECG recordings in the training set or test set. The “Normal heartbeat” and “PVC” represent the numbers of regular heartbeats and PVC in the training set or test set.

**Table 6:** Three types of pooling operations.

Type	Operation
Max-pooling	The maximum pixel value of the batch is selected
Min-pooling	The minimum pixel value of the batch is selected
Average-pooling	The average value of all the pixels in the batch is selected

Here, the “batch” means a group of features that are the overlapping parts of these two vectors: The pooling layer’s kernel and the input vector.

**Table 7:** The parameters related to the experiment.

Batch Size	Number of Features	Margin	Distance	Epsilon	Optimizer	LR	WD	*K*
32	32	0.2	Cosine Similarity	0	Adam	0.0001	0	1

**Table 8:** The performance of applying different noise reduction algorithms on the proposed method.

Noise Reduction Algorithms	Acc (%)	Se (%)	Sp (%)	P_+_ (%)	P_−_ (%)	Time
None	99.63	96.74	99.85	97.97	99.76	0.00
FIR filters	99.56	96.66	99.78	97.04	99.75	0.23
Median filters	99.53	96.9	99.73	96.37	99.77	6.58
FIR filters and median filters	99.46	96.15	99.71	96.12	99.71	7.04

Here, the “Time” means the time it takes to denoise a half-hour ECG.

**Table 9:** The results of the varying number of features.

The Number of Features	TN	FP	FN	TP	Acc (%)	Se (%)	Sp (%)	P_+_ (%)	P_−_ (%)
2	33,808	60	91	2457	99.59	96.43	99.82	97.62	99.73
8	33,800	68	89	2459	99.57	96.51	99.8	97.31	99.74
32	33,817	51	83	2465	99.63	96.74	99.85	97.97	99.76
64	33,802	66	84	2464	99.59	96.7	99.81	97.39	99.75

**Table 10:** The detailed results of testing the pooling layer.

Pooling Type	Acc (%)	Se (%)	Sp (%)	P_+_ (%)	P_−_ (%)
Max-pooling	99.63	96.74	99.85	97.97	99.76
Average-pooling	99.59	97.49	99.74	96.62	99.81

**Table 11:** The experiment results about the margin and epsilon.

Margin	Epsilon	TN	FP	FN	TP	Acc (%)	Se (%)	Sp (%)	P_+_ (%)	P_−_ (%)
0.1	0.0	33,824	44	65	2483	99.70	97.45	99.87	98.26	99.81
0.2	0.0	33,817	51	83	2465	99.63	96.74	99.85	97.97	99.76
0.4	0.0	33,812	56	69	2479	99.66	97.29	99.83	97.79	99.80
0.8	0.0	33,786	82	49	2499	99.64	98.08	99.76	96.82	99.86
0.1	0.1	33,808	60	78	2470	99.62	96.94	99.82	97.63	99.77
0.1	0.2	33,787	81	64	2484	99.60	97.49	99.76	96.84	99.81
0.1	0.3	33,795	73	70	2478	99.61	97.25	99.78	97.14	99.79

**Table 12:** The performance of the KNN classifier with different *K* values.

K	TN	FP	FN	TP	Acc (%)	Se (%)	Sp (%)	P_+_ (%)	P_−_ (%)
1	33,824	44	65	2483	99.7007	97.449	99.8701	98.2588	99.8082
3	33,824	44	66	2482	99.6979	97.4097	99.8701	98.2581	99.8053
5	33,825	43	68	2480	99.6952	97.3312	99.873	98.2957	99.7994
9	33,822	46	69	2479	99.6842	97.292	99.8642	98.1782	99.7964
11	33,822	46	70	2478	99.6815	97.2527	99.8642	98.1775	99.7935

## Data Availability

The data presented in this study are openly available in [physionet] at [10.1109/51.932724 and 10.1161/01.cir.101.23.e215], reference number [38,39]. The webpage of the MIT-BIH Arrhythmia Database is “https://www.physionet.org/content/mitdb/1.0.0/” (accessed on 22 February 2021).
